# Efficient population coding of naturalistic whisker motion in the ventro-posterior medial thalamus based on precise spike timing

**DOI:** 10.3389/fncir.2015.00050

**Published:** 2015-09-25

**Authors:** Michael R. Bale, Robin A. A. Ince, Greta Santagata, Rasmus S. Petersen

**Affiliations:** ^1^School of Life Sciences, University of SussexBrighton, UK; ^2^Faculty of Life Sciences, University of ManchesterManchester, UK; ^3^Instituto de Neurociencias Alicante UMH-CSICSant Joan d’Alacant, Spain; ^4^Institute of Neuroscience and Psychology, University of GlasgowGlasgow, UK

**Keywords:** vibrissa, VPM, neural coding, information theory, synchrony, somatosensory

## Abstract

The rodent whisker-associated thalamic nucleus (VPM) contains a somatotopic map where whisker representation is divided into distinct neuronal sub-populations, called “barreloids”. Each barreloid projects to its associated cortical barrel column and so forms a gateway for incoming sensory stimuli to the barrel cortex. We aimed to determine how the population of neurons within one barreloid encodes naturalistic whisker motion. In rats, we recorded the extracellular activity of up to nine single neurons within a single barreloid, by implanting silicon probes parallel to the longitudinal axis of the barreloids. We found that play-back of texture-induced whisker motion evoked sparse responses, timed with millisecond precision. At the population level, there was synchronous activity: however, different subsets of neurons were synchronously active at different times. Mutual information between population responses and whisker motion increased near linearly with population size. When normalized to factor out firing rate differences, we found that texture was encoded with greater informational-efficiency than white noise. These results indicate that, within each VPM barreloid, there is a rich and efficient population code for naturalistic whisker motion based on precisely timed, population spike patterns.

## Introduction

Thalamus is the gateway to the cerebral cortex and the manner in which sensory signals are encoded by thalamic populations fundamentally constrains cortical computation (Sherman and Guillery, 2009). Although it has long been suspected that neuronal activity within modular, neuronal populations is crucial for thalamo-cortical processing, few studies have simultaneously recorded the activity of multiple neurons within such modules in the thalamus (Desbordes et al., 2008; Temereanca et al., 2008; Wang et al., 2010b). The whisker system is ideal for investigating thalamic population coding (Petersen et al., 2009), since there is a well-defined, modular population of neurons devoted to each whisker at each of the major stations of the sensory pathway (Woolsey and Van der Loos, 1970). In the ventro-posterior medial nucleus (VPM) of the thalamus, each whisker is primarily represented by a cluster of ~250 neurons (in rat) known as a “barreloid” (van der Loos, 1976).

In the whisker system, mechanoreceptors embedded in the whisker follicle-sinus complex project to the cerebral cortex via parallel, topographic pathways through brainstem and thalamus (reviewed by Diamond and Arabzadeh, 2013). The major (lemniscal) pathway, primarily responsible for encoding whisker-object touch, involves VPM (Deschênes et al., 2005). The core of VPM receives ascending input from the principal nucleus of the brainstem and projects primarily (although not exclusively) to layer IV of primary somatosensory cortex (S1) with collaterals to the thalamic reticular nucleus (NRT). Analogously to other sensory systems, VPM conveys ascending sensory signals to cortex and modulates those signals depending on cortical and behavioral state (Temereanca et al., 2008; Sherman and Guillery, 2009). VPM activity is modulated by input from S1 layer 6, NRT and brainstem neuromodulatory centers. VPM is neurochemically more homogeneous than other thalamic nuclei, such as LGN, in being devoid of GABAergic interneurons and consisting of glutamatergic, S1-projecting relay neurons.

By recording the activity of individual neurons, previous studies have determined that VPM neurons can respond to whisker deflection with high (sub-millisecond) spike timing precision and have identified sensory features to which VPM neurons respond (Waite, 1973; Simons and Carvell, 1989; Pinto et al., 2000; Brecht and Sakmann, 2002; Castro-Alamancos, 2002; Yu et al., 2006; Montemurro et al., 2007a; Petersen et al., 2008; Ego-Stengel et al., 2012). At the population level, it is known that sudden whisker contact (e.g., a ramp-and-hold stimulus) evokes a response where different neurons fire within a few milliseconds of each other—commonly termed “synchrony”—and that the degree of synchrony varies with stimulus features (Pinto et al., 2000; Temereanca et al., 2008; Wang et al., 2010b). However, it is not known how VPM neurons, at either single neuron or population level, respond to naturalistic whisker motion. Coding principles for naturalistic stimuli can differ substantially from those for artificial stimuli (Mainen and Sejnowski, 1995; de Ruyter van Steveninck et al., 1997). Our aim here was to investigate how the population of neurons in an individual barreloid of the VPM nucleus encodes texture-induced motion of the corresponding whisker and to test whether its population response is synchronous.

To address these issues, we used multi-microelectrode arrays to record simultaneously the activity of multiple single units from within a single VPM barreloid, in response to play-back of texture-induced whisker motion (Arabzadeh et al., 2005; Wolfe et al., 2008; Lottem and Azouz, 2011; Bale et al., 2013). We found that naturalistic whisker motion evoked precisely timed patterns of population spiking, where different groups of neurons fired synchronously at different times (“dynamic synchrony, DS”). Furthermore, mutual information scaled linearly with population size, suggesting a marked lack of redundancy, and responses exhibited a greater informational efficiency for naturalistic stimuli.

## Materials and Methods

### Electrophysiology

All experimental procedures were approved both by the UK Home Office and by The University of Manchester Ethical Review Committee. Adult male Wistar rats (*n* = 13, 250–350 g) were anesthetized with urethane (1.5 g/kg body weight) and placed into a stereotaxic apparatus. A craniotomy was made over the hemisphere ipsilateral to whisker stimulation 2.0–4.5 mm posterior and 1.5–4.0 mm lateral to bregma. A single-shank, 32-site silicon probe (single row of recording sites, each area 177 μm^2^, 50 μm spacing; recording site impedance lowered by Iridium oxide activation, top and bottom sites used to pass current for marking recording site; Neuronexus, Ann Arbor, MI) was inserted into the brain at 45° to the midline in the coronal plane (dorso-medial to ventro-lateral), to target the contralateral VPM nucleus. During the experiment, position of the probe within VPM was verified by short-latency responses evoked by manual stimulation of the whiskers. The probe was lowered until whisker-evoked activity ceased, and receptive fields characteristic of the ventroposterior lateral nucleus (VPL) emerged on the deepest recording sites (mean 8.3, SD 0.3 mm from pial surface). Extracellular signals were preamplified, digitized at 24.4 kHz, band pass filtered (300–3000 Hz) and continuously stored to hard disk for offline analysis.

### Whisker Stimulation

All whiskers were trimmed to 5 mm and receptive fields were mapped for each recording site by manually deflecting single whiskers. The whisker that evoked responses at the greatest number of recording sites was selected for stimulation. This whisker was inserted into a pulled pipette tube attached to a piezoelectric actuator (P/N PL127.10; Physik Instrumente) positioned 1 mm from the face. The actuator was shortened to raise resonant frequency and fixed to an aluminium tube via small plastic screws. The dynamic range of the actuator was 0.8 mm (whisker deflection range ~40°).

We employed two types of dynamic whisker stimulation: low-pass filtered (300 Hz) white noise (Petersen et al., 2008) and a naturalistic texture stimulus (Bale et al., 2013). The texture stimulus was constructed from optical measurements of whisker motion made from awake rats by Wolfe et al. (2008). Wolfe et al. (2008) trained rats to whisk a textured surface (sandpaper: P150, P400, P800 or P1200) and used a CCD array to measure traces of texture-induced whisker motion in the rostro-caudal plane. In order to construct the stimulus corresponding to a given grade of sandpaper, we considered only periods of the CCD traces during which whiskers were in contact with that sandpaper. Such traces were stitched together so that the final position of one trace equalled the first position of the subsequent one until a sequence of 10 s duration was obtained. To minimise potential contribution of head movement, the resulting sequence was high-pass filtered (FIR at 1 Hz). Finally, to avoid mechanical resonance from the actuator, the sequence was low-pass filtered (FIR 600 Hz). This procedure was repeated for each of the four grades of sandpaper.

The stimulus protocol consisted of 50–100 trials. Each trial consisted of a fixed 10 s sequence of white noise and 1–4 sequences of 5–10 s repeated texture motion. Stimulus sequences were presented in a randomised order and were separated by 1 s intervals without whisker motion. A schematic for an example session (in which a single texture sequence was presented) is shown in Figure 2A.

For consistency with the conditions under which the whisker motion data were registered, all whisker motion stimuli were delivered in the rostro-caudal direction. Accurate reproduction of stimulus position, velocity and acceleration was confirmed by optical testing with a phototransistor circuit (Storchi et al., 2012).

### Histology

At the end of each experiment, two lesions were made by passing 20 μA for 6 s through both the deepest and most superficial sites on the silicon probe. As detailed in Montemurro et al. (2007a), animals were perfused transcardially with saline and formalin. Brains were removed, immersed in fixative for at least 1 day and then transferred to a phosphate-buffered 30% (w/v) sucrose solution for a further 48 h. Coronal sections of 50 μm were stained with cresyl violet. Recovery of the lesion sites confirmed that all single units reported in this study were located in VPM.

### Spike Sorting

**Figure 1:**
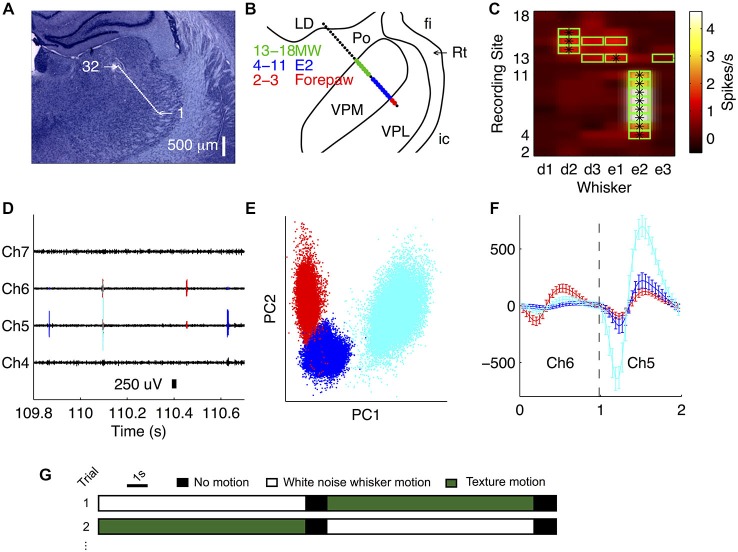
**Recording from VPM using multi-site silicon probes. (A)** Location of recording sites (32-channel silicon probe, sites spaced by 50 μm) in an example recording. Nissl stained coronal section (3.4 mm posterior to bregma). **(B)** Receptive field map obtained during experiment, superimposed on thalamic nuclei reconstruction. LD, lateral nucleus; fi, fimbria; Rt, thalamic reticular nucleus; ic, internal capsule; VPL, ventroposterior lateral nucleus. MW, multi-whisker. **(C)** Multi-unit activity (MUA) evoked by stimulation of each of six whiskers at each recording site. Green outline signifies units with evoked firing rate significantly greater than spontaneous, consistent with online receptive field mapping multi-whisker (MW) observations in **(B)**. *Denotes the principal whisker (PW) at each recording site. **(D)** Unit identities overlaid on an example trace of the raw recording from the experiment shown in **(B)**. **(E)** Waveforms for all identified spikes projected onto the first two principal components of their multichannel waveforms; each color represents a different unit (matches **D**). **(F)** Average single unit waveforms for three extracted clusters on two different channels from the recording shown in **(B)**. **(G)** Schematic illustrating the experimental protocol.

The first step of our data analysis was to isolate single unit activity from the extracellular recordings. We found that spikes emitted by a given single unit were typically recorded from 1–3 recording sites (three recording sites span 100 μm). To exploit this information for better single unit isolation, and also to prevent potential double counting of spikes on adjacent channels, we spike sorted 1–3 channels simultaneously (Figures 1D–F). We extracted 1 ms segments around voltage-threshold-crossing times, concatenating segments from 1–3 adjacent recording sites and clustered these data in the space of their principal components using a *t*-distribution mixture model (Figure 1E, Shoham et al., 2003; Bale and Petersen, 2009). Only clusters exhibiting a clear refractory period were accepted. To test for potential double counting of spikes isolated from adjacent blocks of recording sites, we checked for suspicious peaks (width 0.08 ms, corresponding to two sampling intervals) in the cross-correlation function (bin size = 0.04 ms). Any units contributing to such peaks were excluded. An example recording of four simultaneously recorded adjacent channels is shown in Figure 1D, of which two sites were sorted together (channels 5 and 6). The spikes extracted during the sorting routine (from Figure 1F) are overlaid.

### Localization of Receptive Fields

During each recording, we identified the whisker that evoked the greatest multi-unit activity (MUA) on each recording site (its principal whisker, PW) to manual deflection of individual whiskers. To assess the multi-unit PW quantitatively, we then recorded responses to piezoelectric deflection of the PW in both caudal and rostral directions with a ramp-and-hold protocol. This was repeated for each of the surrounding whiskers. In off-line analysis, we computed the MUA spike count evoked by caudal and rostral deflection at each recording site (as detailed above, time window 20 ms), for each whisker. For each site, we determined the whisker that evoked the greatest MUA response. Provided that this was significantly greater than the spontaneous spike count (time window 20 ms; Wilcoxon signed ranks), this was deemed the PW. By repeating this analysis for each recording site, the PW at each site with significant whisker response was obtained (Figure 1C).

The majority of single units (91%) had a receptive field that matched the multi-unit receptive field of the channel to which it was most closely located. The remaining 9% of single units had a receptive field that corresponded to the MU activity of an adjacent whisker.

### Spike Train Sparseness

Sparse codes are metabolically efficient, in that much information is conveyed with few spikes, and are prominent in the brain (Simoncelli and Olshausen, 2001). We measured temporal sparseness (“lifetime sparseness”), using a slight variant of a standard measure (Rolls and Tovee, 1995):

(1) $$S=1-\frac{{\langle r_{t}\rangle }^{2}}{\langle r_{t}^{2}\rangle }$$

Here *r_t_* is the value of the peri-stimulus time histogram (PSTH) in time bin *t* and the angled brackets denote the mean over time bins (bin size 10 ms). If *r_t_* is constant (non-sparse response), *S* = 0. In contrast, if *r_t_* is zero except for a few bins (highly sparse response), *S* tends to 1 in the limit of many bins. The original Rolls-Tovee measure, *a*, is related to the one used here through the relation *S* = 1−*a*.

### Stimulus Sparseness

An important characteristic of sensory stimuli is whether or not they are Gaussian-distributed. This is usefully assessed by measuring “sparseness” through the following, standard index (Hyvärinen et al., 2009). Given samples *x* of a sensory signal:

(2) $$S_{stim}=-\sqrt{\frac{\pi }{2}}\frac{\langle \left|x-\mu \right|\rangle }{\sigma_{x}}$$

Where μ is the mean, σ_*x*_ is the standard deviation of *x*. *S_stim_* = −1 for Gaussian distributions; S >−1 for distributions with a thinner peak around the mean and fatter tails (“sparse”).

### Unit Responsiveness

To determine whether a given unit was responsive to the stimulus (white noise or texture), the unit’s spontaneous firing rate was measured as the spike count in the 0.5 s interval prior to stimulus onset and its evoked firing rate was measured in each successive 0.5 s interval throughout the course of the stimulus. A unit was classified as “responsive” if its firing rate in any of the post-stimulus windows was significantly higher than that in the pre-stimulus window (Wilcoxon signed ranks, *p* < 0.001; Bonferroni corrected for multiple comparisons). Non-responsive units were not considered in later analysis.

### Jitter Analysis

**Figure 2:**
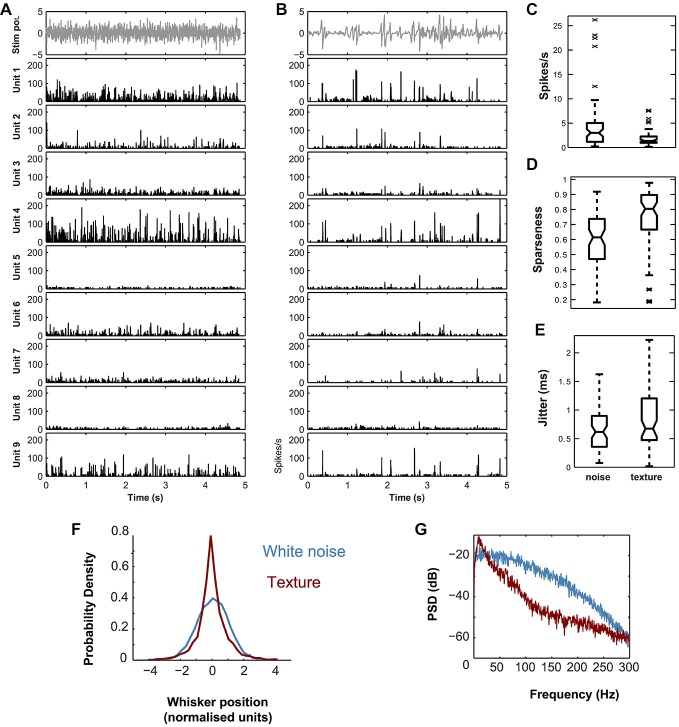
**Response properties of simultaneously recorded single VPM units located within a single barreloid to dynamic whisker stimuli. (A)** White noise stimulus (top panel) and corresponding evoked PSTHs for nine simultaneously recorded units from the same barreloid (lower panels). **(B)** P150 texture stimulus (top panel) and corresponding evoked PSTHs for the same nine units as in A. PSTHs computed with 0.08 ms bins, and smoothed by convolution with a Gaussian (SD 1 ms). Units ordered by recording location from dorso-medial (top) to ventro-lateral (bottom). **(C)** Distribution of single cell firing rates evoked by white noise compared to that evoked by the sandpaper texture stimulus. **(D)** Corresponding plot for sparseness. **(E)** Corresponding plot for spike timing precision (jitter). In **(C–E)** central mark shows median; box edges show 25th, 75th percentiles; whiskers show range of points not considered outliers (approx. ±2.7 SD); crosses show outliers. In panel **(C)**, outliers are not shown. **(F)** Probability Density Function (PDF) of piezo position (normalized units) for the white noise (blue) and texture (red) stimuli. **(G)** Power Spectral Density (PSD) of the same.

Since spike timing precision is a limit on a neuron’s capacity to transmit information, it is necessary to quantify it (Petersen et al., 2009). To quantify spiking precision, we measured the trial-to-trial variability in spike timing (“jitter”) for each unit, as previously described (Montemurro et al., 2007a; Bale et al., 2015). First, we divided the evoked response into 0.8 ms time bins and averaged over trials to form a PSTH, which was then smoothed with a Gaussian filter (SD 1.6 ms). Firing episodes corresponded to local peaks in the PSTH. To focus on reliable episodes we selected peaks which satisfied the following two conditions for the jitter calculation: (1) firing rate was at least half of the maximum for that unit and (2) firing rate exceeded a threshold set according to the null hypothesis that the unit fired randomly at the same average rate (*p* = 0.001). To establish the second condition, we repeatedly simulated random spike trains from a homogeneous Poisson process with the same rate as the time-averaged firing rate of the considered unit. The threshold was set as the 99.9th percentile of these peak heights. For each peak meeting both criteria, we extracted all spikes fired within ±10 ms of the time of the peak and computed spike times relative to the peak time. These time differences were pooled across both trials and peaks and the resulting distribution fitted to a Gaussian. Jitter was defined as the SD of this Gaussian. A minority of units were only weakly modulated by the whisker stimulus and did not exhibit well-defined firing episodes, resulting in high and unreliable values of calculated jitter. In the jitter plot (Figure 2E), we do not plot the points which lie outside the whiskers (defined as median ± 2.7 SD). There were seven such points for white noise and 10 for texture out of a total of *N* = 65 single units.

### Synchrony Analysis

Synchrony is an interesting property of neuronal population activity, since it can enable reliable transmission of information even if individual synapses are weak (Bruno and Sakmann, 2006). As a first test of synchrony, for each pair of simultaneously recorded units, we computed the cross-correlation function for the stimulus-evoked activity on each trial and then averaged the data across trials (binsize 1 ms). This was done separately for the white noise and texture stimuli. For each pair of units, we calculated the peak value of the trial-mean cross-correlation function (the “CCG peak”) as well as its standard error. A unit pair was defined as having a significant cross-correlation peak if the CCG peak exceeded at least three standard errors.

To test for synchrony in our simultaneously recorded units, we registered the response of each unit in 10 ms time bins, and computed the number of bins in which *n = 2, 3, 4 …* units all fired. As a control, we compared this to the number of bins in which such events would be expected by chance from a statistically independent population of units, with the same single unit spike count distributions. To do this, we shuffled the response of each unit independently across time bins. This preserved the overall firing rate and response diversity, while removing the temporal structure. We then computed the same synchrony measure (number of bins in which different numbers of multiple units fired) for this surrogate data set.

With the simplest (static) type of synchrony, each evoked synchronous population response has the same distribution of population response words (that is, the same set of units fires together). Alternatively, different evoked response events might have different distributions of response words. We term this dynamic synchrony (DS), since events at different times evoke different patterns of synchrony. To quantify the extent to which synchrony is dynamic we used an information theoretic approach. We first isolated synchronous population firing events by identifying time bins (20 ms) where two or more units fired on at least 5% of trials. We quantified the degree of DS as the mutual information between the population response words and the peri-stimulus time of the the response event [Ish information estimator (Montemurro et al., 2007b), Panzeri-Treves bias correction (Panzeri and Treves, 1996)]. To normalize for the potential effect of events of different strengths eliciting the same patterns of synchronous activity but with different probabilities, we consider in the information calculation only trials where at least one unit fired. This is equivalent to removing the response word [0, …, 0] from the probability distribution and renormalizing appropriately. If all synchronous population events elicit the same pattern of synchronous activity the information computed in this way should be zero. If it is not, this shows that different synchronous population events evoke different distributions of response patterns—hence the synchrony is dynamic. This can be interpreted as a model comparison between a model where all events elicit population response words from the same distribution, and one in which each event elicits responses from a different distribution—a different pattern of synchrony. To determine the significance of the observed information values we use a permutation testing approach. The null hypothesis for this test is that the observed synchrony is static; the same distribution of response words is evoked by each event (albeit with possibly different pooled firing rate). We therefore performed 1000 permutation calculations in which the identity of the events that elicited each response were randomly shuffled. Mutual information values are affected by the number of trials as well as the size of the spaces considered. Since different recordings had different numbers of trials, cells and detected events it is therefore difficult to compare information values directly. To address this we standardized (*z*-scored) the observed information for each recording with respect to the corresponding set of permutation values. This gives a measure of the statistical strength of the result in favor of DS, and provides a measure in standardised (*z*-score) units that can be compared across different experiments.

### Mutual Information Analysis

Mutual information is a powerful, non-linear measure of the correlation between two variables. Applied to neural coding, it quantifies the intuitive notion of the “information” that a neuron (or neurons) conveys about a stimulus. A key advantage is that it makes minimal assumptions on the functional relationship between the encoded parameter (e.g., whisker position) and the neuronal response; a second advantage is that it provides a rigorous yardstick for comparing alternative, candidate neural codes. Mutual information can give useful physiological insight concerning whether, for example, precise spike timing increases the capacity of a neuron to transmit sensory messages or whether different neurons in a population convey the same messages (redundancy) or complementary ones. Application of information theoretic methods to the whisker system has been reviewed elsewhere (Petersen et al., 2009; Ince et al., 2010).

The aim of this analysis was to quantify how much total information a given population response code conveys about the dynamic whisker stimulus. To this end, we used the spike trains evoked by the repeated whisker motion sequences. We employed the well-established method of Strong et al. (1998) and conducted the analysis for simultaneously recorded populations of single units. For a population of units *i* = 1, …, *N*, we computed the response ***r*** as the *N*-element binary word (*r*_1_, *r*_2_, …, *r_N_*) within the time bin (*t*, *t* + 10 ms). Here, r_*i*_ denotes the (binary) response of unit *i* where 0 indicates that the unit was inactive (no spikes emitted) in that response bin, and 1 indicates that the unit was active (at least 1 spike emitted); *t* denotes time with respect to stimulus onset (*t* = 0). In this approach, these time bins are each considered as the response to a particular stimulus; the stimulus set S is then the set of response bins (implicitly, the small segments of dynamic stimulus preceding each response bin). In this way the explicit consideration of particular stimulus features is replaced by the temporal exploration of a range of inputs from an approximately ergodic process. The method therefore takes into account, without any prior assumptions, all possible stimulus features that might be driving the neural response. The particular number of stimuli available depends on the length of the repeated stimulus segment considered as well as the bin size used. The resulting information value quantifies how discriminable, on average, different response bins (and hence the different preceding stimulus segments) are from each other.

The measurements of *r* were used to estimate the probability *P*(*r*|*t*) of the neural response *r* in time bin, *t*, and the probability *P*(*r*), the mean of *P*(*r*|*t*) over all time bins. When a neural response is reliably modulated by a stimulus, it evokes similar responses across repeated trials at a given time, and different responses across different time bins: thus, *P*(*r*|*t*) varies systematically with *t*. In contrast, when a neuron is insensitive to a stimulus, similar responses are produced for all time bins and *P*(*r*|*t*) is similar to *P*(*r*). *I(R; S)* quantifies how much, on average, *P*(*r*|*t*) differs from *P*(*r*):

(3) $$I(R;S)={\langle \sum_{r\in R}P(r|t)\log_{2}\frac{P(r|t)}{P(r)}\rangle }_{t}$$

Here the angled brackets denote an average over all stimulus time bins. *I(R; S)* quantifies how well, on average, an ideal observer could decide which stimulus time bin was presented from observation of the neural response *r* on a single trial. *I(R; S)* has units of bits; one bit of information indicates that, on average, the uncertainty about which stimulus bin was presented is reduced by a factor of two after observation of a single response.

For each experimental session consisting of *N* units, we computed information values for all possible subpopulations of every size, including single unit information values. For each (sub)-population, the sum of these single unit information values for the population members gives the amount of information that would be conveyed by if the units were transmitting information independently (Averbeck et al., 2006), denoted *independent*. We measured the synergy/redundancy as the difference between the information conveyed by the population and the sum of the single unit information values (Schneidman et al., 2003; Latham and Nirenberg, 2005):

(4) $$Syn(R_{1},R_{2};S)=I(R_{1},R_{2};S)-I(R_{1};S)-I(R_{2};S)$$

Positive values of *Syn*(*R*_1_, *R*_2_; *S*) indicate synergy; the two response variables carry more information about the stimulus together than they do alone. Negative values of *Syn*(*R*_1_, *R*_2_; *S*) indicate redundancy; the two variables together carry less information then the sum of their individual contributions. To test whether a low synergy/redundancy value for a given population might reflect cancelation between strongly redundant and strongly synergistic unit pairs, we conducted the following analysis. For a given population, we computed the synergy/redundancy of all of its constituent unit pairs. Positive (synergistic) and negative (redundant) values were summed separately, resulting in a net pairwise synergy value and a net pairwise redundancy value. The maximum absolute value of these two was taken an indicator of the maximum possible pairwise interaction effect for the population. We then normalized this value with respect to the mutual information conveyed by the population.

To give a more intuitive measure for the mutual information conveyed by a population, we converted bits to bits/s by dividing by the bin width. We also separately normalized for differences in firing rate, by calculating the mutual information per spike (bits/spike), both for single cells and populations.

The probability distributions that appear in equation (3) were computed from a finite number of trials and thus subject to sampling error, which typically inflates estimates of mutual information (Panzeri et al., 2007). Bias correction was performed using the shuffled information estimator, *I_sh_*(*R*; *S*) together with quadratic extrapolation (Montemurro et al., 2007b; Panzeri et al., 2007). Mutual information values were computed using the PyEntropy package (Ince et al., 2009). The length of the repeated stimulus blocks was either 5 s or 10 s, resulting in 500 or 1000 response bins for each information calculation. The crucial parameter for determining the effectiveness of bias correction is the ratio of the number of trials per stimulus to the number of different response symbols (Ince et al., 2010). This number of trials per stimulus is given by the number of repeated presentations of the continuous stimulus and does not change across different stimulus block lengths or response bin sizes. The number of response symbols (2 to the power of the population size) is also fixed, so the difference in stimulus block lengths does not affect the bias of the information measure. With the available data, the number of trials per stimulus was 50–200 (median 200); the ratio of trials/stimulus to number of response symbols (for the largest population size recorded in each experiment) was 0.2–100 (median 18.75). Simulations have shown (Ince et al., 2009) that with the bias correction employed here, a ratio of ~0.25 can be adequately bias corrected: the data here fall within that range. To further test the accuracy of our bias correction, we repeated the analysis with a surrogate data set in which, for each trial, the population responses were shuffled independently across time bins (so that the true information is zero). The resulting mean information values for each population size were expressed as a percentage of the corresponding unshuffled value. In the most demanding case—population size 9—for white noise stimulation, the median was 0.79%; for texture stimulation, the median was 0.85%. This indicates that bias was effectively corrected.

## Results

### Recording the Population Response of a VPM Barreloid to Whisker Motion

Our primary aim was to investigate how the population of neurons within a single VPM barreloid collectively encodes naturalistic whisker motion. To address this, it was essential to deliver multiple, identical repeats of controlled, whisker motion sequences. To this end, we implanted 32-channel silicon probes into the VPM of urethanised rats, and recorded simultaneously the responses of multiple single units to deflection of the whiskers. To concentrate recording sites as far as possible within the same barreloid, we implanted probes at a 45° angle (dorso-medial to ventro-lateral), approximately parallel to the long axis of the barreloids (Figures 1A,B). To assess microelectrode placement, we identified, for each recording site, the whisker that evoked the greatest multi-unit response to ramp-and-hold deflection (Figure 1C; see “Materials and Methods” Section)—the “PW”. In the example of Figure 1, the PW was E2 for sites 4–11, E1 for site 13 and D2 for sites 14–16. Overall (*N* = 16 recordings), up to 11 recording sites shared the same principal whisker (mean 7.4, SD 2.4 range 4–11), indicating that they recorded neural activity from the same barreloid. Since the recording sites were spaced at 50 μm intervals along the probe shank, 11 sites spanned 500 μm. This length is consistent with anatomical estimates of barreloid length in adult rats (Haidarliu and Ahissar, 2001).

A piezoelectric actuator was used to “play back” sequences of dynamic whisker motion (Bale et al., 2013; see “Materials and Methods” Section). A naturalistic stimulus was generated from optically registered whisker sweeps of rats actively whisking sandpaper (Wolfe et al., 2008). To benchmark the coding efficiency of this stimulus, we also used low-pass filtered white noise (hereafter abbreviated to “white noise”; Figure 2A), which was normalized to have the same standard deviation. Both stimuli were applied to individual whiskers (*N* = 16 recordings), and we recorded neuronal responses from all probe sites simultaneously.

### Response of Single VPM Units to Naturalistic Whisker Motion are Temporally Precise and Sparse

Figure 2 shows PSTHs, evoked by white noise (Figure 2A) and texture (Figure 2B) applied to whisker E2, for a set of 9 responsive single units (see “Materials and Methods” Section) recorded simultaneously from barreloid E2. The single unit responses to the two stimuli were qualitatively similar. Both stimuli evoked responses that consisted of temporally isolated firing episodes. We quantified the spike timing precision of these firing episodes by estimating the trial to trial “jitter” in spike time (Figure 2E; see “Materials and Methods” Section). Jitter for both texture and white noise was sub-millisecond (median 0.48 ms, IQR 0.48–1.2 ms and median 0.36 ms, IQR 0.36–0.88 ms respectively). The main differences between the stimuli were that white noise evoked a higher rate of firing events (Figure 2C; median 3.0 spikes/s compared to 1.4 spikes/s; signed rank test, *p* < 1e^−7^), while responses to texture exhibited higher temporal sparseness (Figure 2D; median 0.80 compared to 0.61; signed rank test, *p* < 1e^−9^). To investigate the source of these differences we compared the stimuli. Compared to white noise, the texture stimulus both had a markedly non-Gaussian “sparse” distribution (Figure 2F) and contained less relative power at high frequencies (Figure 2G). Using a standard index which is −1 for a Gaussian and >−1 for sparse distributions (Material and Methods, Equation 2), the white noise had an index value of −1 and the texture −0.85.

### VPM Population Responses Exhibit Dynamic Synchrony

**Figure 3:**
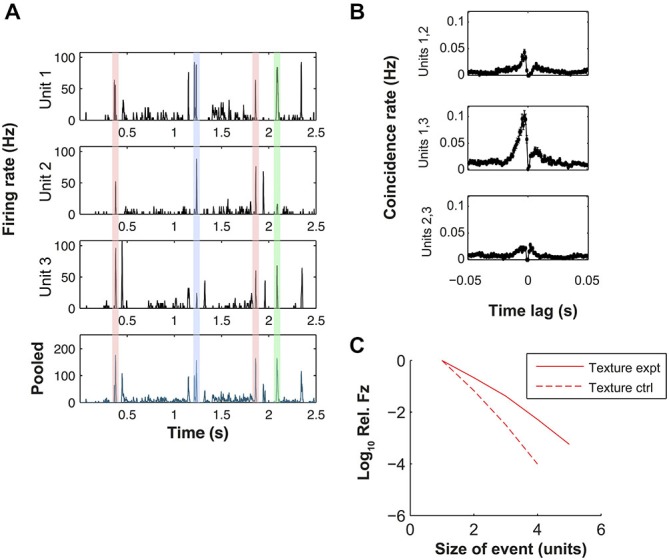
**Dynamic synchrony (DS) evoked by texture stimulus. (A)** Top three plots: PSTHs for three simultaneously recorded units evoked by a sandpaper texture stimulus (units 1–3 here correspond to units 1, 2 and 4 of Figure 2 respectively). Bottom plot: peri-stimulus time histogram (PSTH) of pooled response of the same units. Shaded regions highlight similar peaks in the population PSTH that arise from different patterns of population activity (color coded). **(B)** Cross-correlograms for each pair-wise combination of the units of panel **(A)**. **(C)** Log frequency with which a given number of units fired simultaneously (10 ms bin). Frequency is normalized by the single unit firing rate. Solid line shows results for simultaneously recorded data, dashed line shows control under null hypothesis that each unit fired independently at random (see “Materials and Methods” Section).

Our finding that texture evokes precisely timed spikes raises the question of the nature of the response at the population level. Motivated by previous studies of the VPM response to periodic whisker stimulation (Bruno and Simons, 2002; Temereanca et al., 2008; Wang et al., 2010b), we investigated whether the population response manifested synchrony, conventionally defined as coincident firing on a time-scale of ~10 ms (Wang et al., 2010a). Inspection of the recordings revealed qualitative evidence for synchrony. Figure 3A shows a detailed view of the responses of 3 simultaneously recorded single units (a subset of those shown in Figure 2) evoked by the texture stimulus. Within this 2.5 s excerpt, there were several times at which the stimulus evoked a marked increase in firing rate in 2 or more units (shaded regions).

To quantify the synchrony, we first computed cross-correlograms (CCGs) for the texture-evoked activity (Figure 3B, for units with PSTHs shown in Figure 3A). Eighty four neuron pairs (out of a total of 145) exhibited statistically significant CCG peaks (see “Materials and Methods” Section). For the significant peaks, the CCG peak amplitude was 0.03 ± 0.11 coincidences/spike (median ± SD), the CCG (absolute) peak lag was 2.5 ± 5.8 ms and the CCG peak width (full width at half maximum, FWHM) was 4.6 ± 3.6 ms. Results for white noise were similar: 71 pairs exhibited significant CCG peaks; for these peaks, CCG peak amplitude was 0.08 ± 0.27; lag was 2.0 ± 5.6 ms and CCG peak FWHM was 3.3 ± 5.1 ms.

To extend the analysis of synchrony beyond the pairwise CCGs to neuronal sets of arbitrary size, we used the following procedure. We registered the response of each unit in 10 ms time bins, and computed the number of bins in which *n = 2, 3, 4 …* units all fired on the same trial. As a control, we compared this to the number of such events expected from random firing (see “Materials and Methods” Section). We found, for the texture-evoked response of the nine simultaneously recorded units of Figure 2A, that synchronous firing of three and four units occurred more often (14.6 and 66.8 times respectively) than expected from random firing (Figure 3C). Synchronous firing of 5 units occurred in the experimental data but never in the control. On average, across all sets of simultaneous recordings, synchrony occurred more frequently than expected by random firing.

These results indicate that both white noise and texture whisker motion induced synchronous firing within the associated VPM barreloid. However, closer inspection of the data revealed that the precise pattern of synchrony changed over time: different constellations of neurons fired at different times during the stimulus. The shaded regions in Figure 3A illustrate four response episodes where the pooled firing rate of the population was similar but the mean population spike patterns were, in 3 cases, distinct. For two of the peaks all three units exhibited high firing rate at almost the same time (red shading), but at other times different combinations of units responded: units 1 and 2 but not 3 (blue shading) or units 1 and 3 but not 2 (green shading).

With the simplest (static) type of synchrony, each evoked synchronous population response has the same distribution of population response words (that is, the same set of units fires together). Alternatively, different evoked response events might have different distributions of response words. Since events at different times evoke different patterns of synchrony, we refer to this type of synchrony as dynamic (dynamic synchrony, DS). DS implies that there is a repertoire of different synchronous response patterns and that there is a systematic relationship between a particular stimulus event and the evoked response pattern. DS can be quantified by measuring the strength of this association. Specifically, we quantified DS with a normalized mutual information value (see “Materials and Methods” Section). This value can be interpreted as a *z*-score: under the null hypothesis of static synchrony, it should take values drawn from a standard normal distribution. For all seven recordings in which synchronous events were reliably identified (3–9 simultaneously recorded units; 3–121 synchronous events) the DS measure was highly significant (*p* < 0.001; permutation test, *N* = 1000). We found that responses to texture stimuli resulted in a DS measure of 35.8 ± 21.1 (standardised units) while for those to white noise, the values were 26.8 ± 23.1; but that this difference was not significant (Wilcoxon signed rank test, *p* > 0.3).

### VPM Populations Encode Information Independently

**Figure 4:**
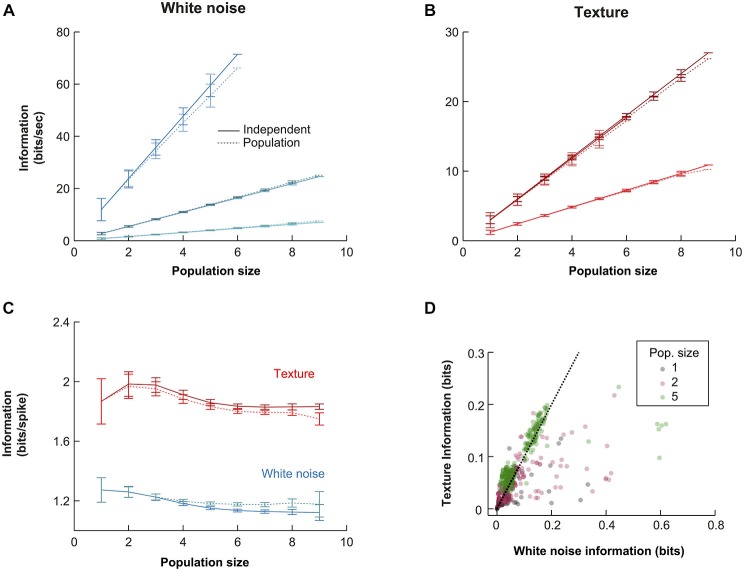
**Quantitative comparison of alternative population codes. (A)** Mutual information conveyed about the white noise whisker stimulus by the VPM population (bin size 10 ms) for the three experiments with the largest number of simultaneously recorded units. “Population” denotes the information in the synchronous population response; “Independent” denotes the linear sum of the information conveyed by each individual neuron within the considered population. Each point is an average over all possible sub-populations of a given size (error bars show SEM over populations). **(B)** Corresponding results for responses to sandpaper texture stimulus. **(C)** Information conveyed by the different codes under white noise (blue) and texture (red) stimulation for all subpopulations obtained over all experimental sessions. Information values are normalized by the total firing rate of each considered sub-population to obtain units of bits/spike. Each point is the mean over all sub-populations of that size; error bars show SEM. **(D)** Scatter plot of raw information values for single units, pairs of units and populations of size 5.

To guide our investigation of the population response, we examined how the information conveyed by the population scaled with population size. Such an analysis can reveal whether the representation is, overall, redundant (sub-linear scaling), independent (linear scaling) or synergistic (super-linear scaling). We estimated the mutual information that the population spike pattern (the simultaneous response of all neurons in a given time bin) conveyed about white noise and texture (see “Materials and Methods” Section). In all cases, we found mutual information to scale approximately linearly with population size. We illustrate this with the three experiments with highest numbers of simultaneously recorded units (Figure 4). The similarity between the mutual information available in the population and that in the sum of single unit information values, across a range of population sizes (Figures 4A,B, independent solid lines, pattern dotted lines), implies a striking lack of redundancy.

In principle, zero redundancy for a population could mask significant redundancy/synergy in subsets of the population that cancel out. To test this, we first calculated pairwise synergies and redundancies directly (see “Materials and Methods” Section). For each recording with two or more simultaneous units, we calculated the mean absolute synergy/redundancy value (Equation 4) of each unit pair, as a percentage of the mutual information conveyed by that pair. Across recordings (*N* = 11), the mean was 2.1 ± 1.7% for white noise and 1.8 ± 1.0% for textures. This shows that net pairwise interaction effects were small. Second, for recordings in which at least five units were recorded (*N* = 3; those shown in Figure 4A), we considered all possible sub-populations of size 5. For each such sub-population, we computed an index which expresses the maximum possible pairwise synergistic/redundant effect, as a percentage of the mutual information conveyed by the sub-population (see “Materials and Methods” Section). This resulted in a maximum pairwise synergy/redundancy cancellation of 2.0 ± 1.5% (*N* = 258 populations, mean ± SD). While this analysis does not exclude the possibility of potential higher order effects, it does show that the near-zero redundancy observed at the population level was not a consequence of cancelation between synergy and redundancy in pairs of units.

### VPM Populations Encode Naturalistic Texture Motion Efficiently than White Noise Motion

We repeated the information calculations for all recordings (*N* = 16; Figure 4D). To factor out the effect of different firing rates, we normalized the mutual information values to units of bits/spike and then averaged the results over all simultaneous recordings of a given size (Figure 4C). The normalization to units of bits/spike revealed that the mutual information conveyed per spike was higher for the texture stimulus than for the white noise stimulus. At the single unit level (*N* = 65), white noise stimulation yielded mutual information values of 1.33 ± 0.67 [1.29] bits/spike, while texture stimulation yielded 1.94 ± 1.24 [1.64] bits/spike (mean ± SD [median]): this difference was significant (signed ranks test; *p* < 0.00001). For populations of size 6 (*N* = 169), the population code conveyed 1.26 ± 0.15 [1.26] bits/spike with white noise and 1.95 ± 0.26 [1.98] bits/spike with texture (signed ranks test; *p* < 1e^−10^). This difference might mean either that the population transmits more information (in a given period of time) about texture than about white noise or, alternatively, that it conveys the same information but using fewer spikes. The scatter plots in Figure 4D show that the mutual information values conveyed by the response in each 10 ms bin were usually similar for the two types of stimuli (although a proportion of population responses were more informative about white noise). Therefore, the lower firing rate evoked by texture (Figure 2C) accounts for the finding that texture conveys more bits/spike. The population response conveys about the same amount of information about texture motion as white noise, but does so with fewer spikes. This suggests that the subcortical pathway may be adapted to whisker motion with the statistical characteristics of natural object interactions.

### Effect of Noise Correlations on the Population Code

Dynamic synchrony might reflect either: (1) properties of individual units or (2) network properties. To discriminate between these possibilities, we calculated the effect of noise correlations on the population code, using information theory (Schneidman et al., 2003; Latham and Nirenberg, 2005; Chicharro, 2014). Noise correlations are correlations between the responses of simultaneously recorded single units that are not related to, or driven by, the external stimulus; arising instead from intrinsic network activity. The effect of noise correlations can be removed by creating a surrogate data set in which the responses of each unit to a given stimulus are shuffled across trials, with respect to the other members of the population. This surrogate data set is then a model for what the responses would look like if the cells were firing independently. We generated such surrogate data and repeated both the DS analysis and the mutual information based analysis. We found, for both types of stimulus, that shuffling made little difference to the DS measure: the ratio of the shuffled to the unshuffled DS measure was 1.02 [0.82 1.19] (median [min max] across recordings) for white noise and 0.99 [0.90 1.13] for texture. To assess the impact of noise correlations on mutual information, we computed the difference between the unshuffled and shuffled information, normalized by the unshuffled information. Again, for both types of stimulus, the impact of noise correlations was small: 4.1% [2.4 6.7] for white noise, 2.9% [1.8 4.8] for textures (median [min max] across population sizes). These results indicate that the results reported above reflect the coding properties of individual units rather than network interactions.

### Robustness of Results to Differential Parameters of the Mutual Information Estimation

**Figure 5:**
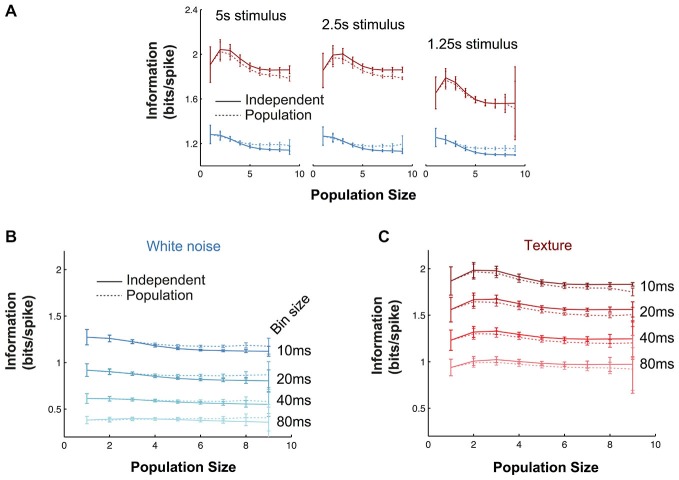
**Robustness of information theoretic analyses. (A)** Mutual information results over all experiments as a function of population size (details as in Figure 4C) when the calculations are performed with stimulus windows of 5, 2.5 and 1.25 s (500, 250 and 125 stimuli respectively). **(B,C)** Mutual information results over all experiments as a function of population size (details as in Figure 4C) for a range of response bin sizes (10–80 ms) for the white noise **(B)** and texture **(C)** stimuli.

It is necessary to test whether estimates of mutual information are accurate (see “Materials and Methods” Section). To assess the robustness of the main mutual information results to the various parameters of the information theoretic analyses, we performed the following controls. First, we investigated the effect of the length of stimulus. In the full data set, stimulus lengths ranged from 5–40 s, corresponding to 500–4000 10 ms bins. To test whether our results were affected by these differences, we repeated the population mutual information analysis using subsections of the stimulus sequence (1.25–5 s) common to all recordings (Figure 5A). In all cases, the texture stimulus evoked more informative responses than white noise (in bits/spike), there was very little redundancy and the values were quantitatively very close to the full results presented in Figure 4C. Information values for the shortest texture stimuli (1.25s) were lower, but we expect this is a consequence of the sparse structure of the stimulus. Second, we tested the effect of bin size used for the information analysis (range 10–80 ms; Figures 5B,C). Again the results were robust: in all cases, texture evoked higher bits/spike than white noise and redundancy was very low. Information values were lower for larger bin sizes; this indicates the temporal precision of the population response is (as expected from the jitter and CCG results) at least 10 ms.

## Discussion

The aim of this study was to investigate how a modular, population of neurons corresponding to one whisker processes complex, naturalistic whisker motion. We focussed on the VPM thalamus, where signals from a given whisker are primarily encoded by a population of ~250 projection neurons within the corresponding barreloid (van der Loos, 1976; Land and Simons, 1985; Sugitani et al., 1990; Haidarliu et al., 2008). We were able to record from up to nine single units within one barreloid simultaneously. In contrast to what has previously been reported with ramp-and-hold stimuli, we found that naturalistic whisker motion evoked a dynamic sequence of population spiking, where different groups of neurons fired synchronously at different times (“dynamic synchrony”).

Previous work on the rat/mouse VPM recorded the activity of 1–2 neurons within one barreloid (Waite, 1973; Simons and Carvell, 1989; Pinto et al., 2000; Brecht and Sakmann, 2002; Yu et al., 2006; Montemurro et al., 2007a; Petersen et al., 2008; Temereanca et al., 2008; Bale and Petersen, 2009; Wang et al., 2010b; Scaglione et al., 2011; Poulet et al., 2012). Here, by using multi-site silicon probes and a novel insertion angle, up to 11 recording sites could be located within a single barreloid. Eleven sites spanned 500 microns, which is consistent with anatomical measurements of barreloid length in adult rats (Haidarliu et al., 2008). This technique enabled us to isolate the activity of up to nine single units within the same barreloid.

“Naturalistic” stimuli, that reflect the complex, dynamic stimuli that animals experience during natural behavior, can evoke responses that differ substantially in reliability and spike timing (Mainen and Sejnowski, 1995; de Ruyter van Steveninck et al., 1997). Although the naturalistic stimulus paradigm is well-established in vision (Simoncelli and Olshausen, 2001), it is only more recently that it has become feasible in the whisker system (Arabzadeh et al., 2005; Lottem and Azouz, 2011; Bale et al., 2013). Our aim here was to study the population response of a VPM barreloid to naturalistic whisker motion. To this end, it was essential to reproduce identical sequences of naturalistic whisker motion on multiple trials. Our approach was to use an anesthetised preparation and to play back sequences of texture-induced whisker motion recorded optically from behaving rats (Wolfe et al., 2008).

Previous work on VPM has shown that rapid whisker motion, such as occurs frequently during a white noise sequence or at the start of a ramp-and-hold stimulus, evokes spikes whose timing is reliable with sub-millisecond precision (Montemurro et al., 2007a). Under some circumstances (periodic whisker deflection), it is known that these spikes occur coincidently across neurons on a time-scale of ~10 ms (“synchrony”; Bruno and Sakmann, 2006; Temereanca et al., 2008; Wang et al., 2010b). We found that naturalistic whisker motion evoked a complex population response where different subsets of neurons are co-active at different times (Figure 3A). In other words, the synchrony was dynamic. We argue that this difference is due to the more complex nature of the stimuli we used. The onset of a ramp-and-hold whisker deflection provokes a correlated increase in position, velocity, acceleration and all higher order temporal derivatives of whisker position (Petersen et al., 2008). Thus, although VPM neurons are tuned to diverse kinetic features (Pinto et al., 2000; Petersen et al., 2008), such a stimulus will tend to evoke a synchronous response from a substantial proportion of the neuron population. Conversely, with a dynamic naturalistic stimulus, the position, velocity and acceleration are decoupled and neurons tuned to different kinetic features tend to fire at different times. Consistent with this interpretation, our quantitative measure of DS was not strongly affected by removing noise correlations from the responses. Moreover, the degree of synchrony exhibited by VPM neuron pairs is known to depend on both deflection velocity and stimulation frequency (Temereanca et al., 2008). Adaptation mechanisms may contribute further dynamism to the response (Wang et al., 2010b). VPM response heterogeneity has also been reported both in the electrical whisking paradigm (Yu et al., 2006) and in the behaving mouse (Gutnisky et al., 2013). These observations are consistent with a model of VPM barreloid function whereby different neurons independently encode different aspects of the on-going whisker motion. Consistent with this, we found that the mutual information conveyed by the population scaled linearly with population size and that this effect was robust to varying the parameters of the analysis. We found no evidence that this lack of redundancy reflected cancelation between strongly synergistic and strongly redundant pairwise sub-populations.

Individual spikes conveyed more bits/spike for the naturalistic texture stimulus compared to white noise (Figure 4C), suggesting that the sensory pathway to thalamus may be optimized towards representing naturalistic stimuli, as has also been suggested in the visual and auditory systems (Dan et al., 1996; Simoncelli and Olshausen, 2001; Lewicki, 2002). Similarly to these other systems, the naturalistic texture stimulus had a notably non-gaussian “sparse” distribution. Compared to white noise, the texture stimulus had relatively greater power at low frequencies. There are likely to be two factors underlying the greater sparseness and informational efficiency of the texture responses: (1) the greater stimulus sparseness and (2) the fact that VPM neurons tend to be velocity-sensitive (Pinto et al., 2000; Petersen et al., 2008) and therefore sensitive to higher stimulus frequency components. Theories of efficient coding (redundancy reduction, predictive coding) predict that, under high signal to noise conditions, there should be little redundancy in the neural representation of a sensory signal (Barlow, 1961; Srinivasan et al., 1982; Atick and Redlich, 1990). There has been extensive investigation of efficient coding in vision (Atick and Redlich, 1990; Simoncelli and Olshausen, 2001; Vinje and Gallant, 2002; Sharpee et al., 2006), a few studies in audition (Smith and Lewicki, 2006; Ming and Holt, 2009) but in somatosensation, the only previous work has been on tactile robots (Hafner et al., 2003; Evans, 2013). As far as we are aware, the current study is the first direct evidence for efficient coding in somatosensation.

Synchrony is potentially important, since it provides a possible explanation for how the thalamocortical pathway might achieve reliable transmission, despite the fact that individual, afferent synapses are typically weak (Bruno and Sakmann, 2006). When a population of thalamic neurons spikes synchronously (on the time-scale of membrane time constant), multiple postsynaptic potentials can summate in a thalamorecipient neuron and trigger postsynaptic spiking. Such an integration mechanism accounts for how stimuli such as ramp-and-hold whisker deflections might trigger a reliable response in thalamorecipient, cortical neurons, and is a potentially effective method of decoding the occurrence and velocity/direction of such stimuli. However, integration is sensitive only to the number of presynaptic spikes and is blind to their origin. It will lose information from a DS code where different patterns of spikes are elicited by different stimulus features. An intriguing possibility is that, through tuning of synaptic strengths (Feldman, 2009) and/or nonlinear dendritic processes (London and Häusser, 2005), cortical neurons are able to perform decoding operations more sophisticated than integration and thereby to discriminate amongst a broad class of sensory signals.

Our findings raise issues for further work. One limitation of the current analysis relates to the information calculations. Mutual information as computed here is a powerful statistic for quantifying, in a general way, the reliability of the population response to a dynamic stimulus. However, it would also be interesting to understand what particular kinematic features of the stimulus drive the population response. Second, although recording from populations of size 9 from a single barreloid is the largest-scale recording yet reported from VPM, some network interaction effects only become appreciable for large population sizes (Roudi et al., 2009), and it would be interesting to achieve still higher population sizes. Finally, it is an important challenge to investigate population coding in the awake, behaving animal. However, since multiple trials of identical whisker motion sequences cannot be delivered under these conditions, the present analysis approaches are inapplicable and new methods will be required.

In sum, our findings suggest that the basic building block of the whisker-related thalamus—the barreloid—encodes naturalistic sensory information in a remarkably efficient manner. Barreloid ensembles exhibit temporally sparse responses that encode rich information about whisker motion through a DS code. The combination of sub-millisecond spike timing precision and diverse tuning leads to a high capacity thalamic population code, which potentially conveys a rich signal about multiple features of whisker motion to cortex. A potentially significant implication of our findings is that it would be advantageous for cortical circuits to act not only as detectors of coincident thalamic activity but also to decode patterns of thalamic population activity.

## Author Contributions

MRB, RAAI and RSP designed the experiment. MRB and GS performed neurophysiology. MRB, RAAI and RSP performed data analysis. MRB, RAAI and RSP wrote the manuscript. MRB and RAAI contributed equally and share first authorship of this manuscript.

## Conflict of Interest Statement

The authors declare that the research was conducted in the absence of any commercial or financial relationships that could be construed as a potential conflict of interest.
